# Cholera

**Published:** 1872-08

**Authors:** 


### CHOLERA

Is characterized by a colorless, odorless, painless, and copious watery discharge from the bowels, leaving a feeling of weakness about the lower limbs, and a sense of falling or giving way in the abdomen, which totally disinclines a person from getting up from either a bed or a chair.

In cholera times, and in a cholera locality, such weakness may come on after an ordinary action. In either case it is not a sign of cholera, — it is cholera itself, and the person should go to bed instantly, in the house or room where he may be at the time; for every single step taken under such circumstances increases the chance of the attack being made fatal, which otherwise might have been recovered from. To attempt to go home, even if but a mile away, a half, or even a third, is a criminal trifling with life. Getting into bed after one of these cholera discharges is an elysium, and the patient almost wishes that nothing may ever occur to make it necessary for him to get up again, so delicious does the rest in bed seem to him. There is present also an intolerable thirst, which admits of no appeasement. Three things are to be done on the instant: Send for a physician; meanwhile crush up between the teeth small bits of ice, swallowing them in as large pieces as possible, and as long as it is agreeable, having taken before the ice a pill made of ten grains of calomel. The effect of this is to stop the purging and vomiting within two hours, giving time for the doctor to arrive, when the whole case should be turned over to him, having given information of all that has been done. The calomel thus arrests the passage promptly, giving time for other action; in the course of a few hours afterwards it gives a bilious passage, and the disease is cured, killed, the patient to be nursed up into health afterwards.

The advantage of this calomel pill is that it has no taste, is quite heavy, and, when it once gets into the stomach, falls to the bottom, dissolves gradually, and nothing can eject it.

It will remain on the stomach when nothing else will. It remains by virtue of its physical quality of heaviness. It is literally as "heavy as lead."

If a pill is not at hand, and there is no means of exact measurement, take an even tea-spoonful of calomel, divide it into ten equal parts, take one part in some soft bread or boiled rice, washing the mouth and teeth particularly well four or five times afterwards, to clear away every particle of mercury, and repeat every two hours until relief or the doctor comes.

If there is a prejudice against calomel, then take the following mixture, which has been found by hundreds of physicians to be a very efficient remedy. It has been so successful here, that it has been sold as a patent medicine, and doubtless will be again, should there be occasion for it. In that event, it is advised that every family have a little of it on hand to use in case of a sudden attack in the night, or failure to have a physician promptly. Three drachms each of laudanum, spirits of camphor, and spirits of turpentine, to which add thirty drops of peppermint. Take one tablespoonful in half a glass of weak brandy and water; that is, a tablespoon of brandy in half a glass of water. Repeat every two hours, if needed.

A more reliable preparation is that by which the Rev. Dr. Hamlin, of Constantinople, saved hundreds of lives when the disease ravaged that city a few years ago. In no case did the remedy fail where the disease could be reached in season. It is no less effective in cholera morbus and ordinary diarrhœa: —

One part laudanum.
One part camphorated spirit.
Two parts capsicum.

Dose, one tea-spoonful in a wine glass of water. If the case is obstinate, repeat the dose in three or four hours.

There is reason to believe that incipient and slight attacks may be cut short by taking equal weights of gum camphor and the best and strongest alcohol, six to twelve drops on lumps of loaf sugar, according to the violence of the attack. Repeat it as often as desired, but under two conditions: Lie still in bed, and, Send for a physician; and if you get well before he comes, so much the better. In cholera times persons from home necessarily, would do well to carry a vial of this mixture along with them.

But it must be remembered that the very foundation stone of safety against cholera, against its spread in city, village, or household, is absolute cleanliness. It can no more spread, it can no more be communicated from one individual to another where there is perfect cleanliness of person and premises, than fire can spread where there is not a particle of air. Therefore —

1. Every householder owes it to himself, to his family, to his neighbors, to the community in which he resides, to have his house, from cellar to garret, from the street curb to the rear line of his lot, most scrupulously cleansed, by sweeping, washing, and whitewashing.

2. Every man who has authority in city or town government should consider himself bound by the oath of office, and by every consideration of humanity, to give himself no rest until every street, alley, close, gutter, and sewer is placed in a state of as perfect cleanliness as possible, and kept so until the frosts of next season come.

3. These cleansings should be done early, in February and March, because, if put off till warm weather, the very effort necessary to the removal of filth will only tend, in the essential nature of things, to hasten the appearance of the disease, to increase its malignity, and to extend the time of its devastations; because the suns of spring and summer the sooner warm into life and intensify the viperic and malignant influence, which in its remorseless tread wrecks so much of human happiness, and desolates so many hearthstones.

In all its long history, the Journal of Health has never advised medicine to be taken in any single case, unless by the direction of an educated physician, except in the case of cholera; but in the nature of things this is exceptional.

